# Single-port laparoscopic adrenalectomy for a right-sided aldosterone-producing adenoma: a case report

**DOI:** 10.1186/1752-1947-6-208

**Published:** 2012-07-18

**Authors:** Akira Sasaki, Shigeaki Baba, Toru Obuchi, Akira Umemura, Masaru Mizuno, Go Wakabayashi

**Affiliations:** 1 Department of Surgery, Iwate Medical University School of Medicine, 19-1 Uchimaru, Morioka, 0200-8505, Japan

## Abstract

**Introduction:**

Single-port laparoscopic adrenalectomy is one of the most interesting surgical advances. Here, we evaluate the safety and feasibility of single-port laparoscopic adrenalectomy as treatment for a right-sided aldosterone-producing adenoma.

**Case presentation:**

A 39-year-old Japanese woman presented with hypertension and hypokalemia. Abdominal computed tomography and an endocrinological workup revealed a 19mm right adrenal tumor with primary aldosteronism. Our patient was informed of the details of the surgical procedure and our efforts to reduce the number of incisions needed - ideally, to a single incision - when removing her adrenal gland. A single-port laparoscopic adrenalectomy was attempted. A multichannel port was inserted through a 2.5cm umbilical incision. A 5mm flexible laparoscope, articulating laparoscopic dissector and tissue sealing device were the primary tools used in the operation. The right liver lobe was evaluated using a percutaneous instrument, providing good visualization of the operative field surrounding her right adrenal gland. The single-port laparoscopic adrenalectomy was successfully completed without any intraoperative complications. The operating time was 76 minutes, and her blood loss was 5mL. Oral intake was resumed on the first postoperative day, and the length of her hospital stay was three days. Her postoperative course was uneventful with no morbidity within one month of follow-up, and our patient had excellent cosmetic results.

**Conclusions:**

Single-port laparoscopic adrenalectomy is a safe and feasible procedure for patients with a right-sided adrenal tumor when performed by a surgeon experienced in laparoscopic and adrenal surgery. However, more surgical experience using this technique is required to confirm our initial impressions.

## Introduction

Since Gagner *et al*. [1] reported the first multiport laparoscopic adrenalectomy (MPLA) in 1992, minimally invasive procedures have become the standard methods used for most patients with benign adrenal tumors [2-5]. Recently, a trial of single-port surgery (SPS) was started because SPS has the potential to provide patients with improved cosmetic outcomes, less postoperative wound pain and fewer wound complications; as such, it satisfies a growing demand for less invasive surgical procedures [6-8]. Now, interest in SPS has greatly increased worldwide. Since January 2010, we have performed single-port laparoscopic adrenalectomies (SPLAs) for 10 patients with benign adrenal tumors. However, the technical challenges of right-sided SPLA have prevented its widespread use. The aim of this report was to evaluate the safety and feasibility of SPLA for the treatment of a right-sided aldosterone-producing adenoma.

## Case presentation

**Figure 1:**
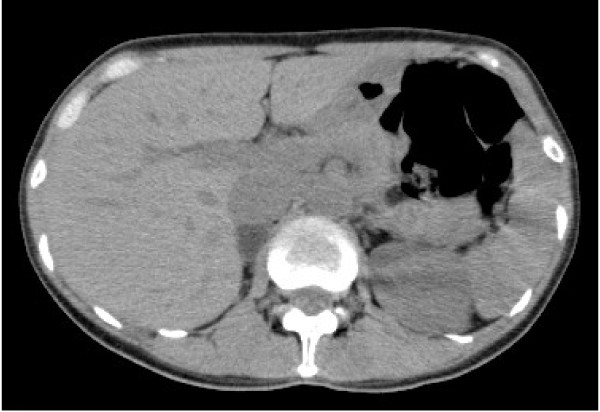
**Abdominal computed tomography showing a 19mm right adrenal tumor**.

A 39-year-old Japanese woman (body mass index of 18kg/m^2^) with a past medical history of hypertension was referred to our hospital because of hypertension and hypokalemia. She had a history of a Cesarean section at 30 years old. A 19mm right adrenal tumor was found on abdominal computed tomography (Figure 1). Bilateral adrenal vein catheterization was performed to obtain plasma samples from both her adrenal veins and infrarenal inferior vena cava. The basal aldosterone levels in her right and left adrenal vein were found to be 51,900pg/mL and 1,200pg/mL, respectively. Thirty minutes after stimulation with adrenocorticotropic hormone, these levels rose to 113,000pg/mL and 1,5100pg/mL, respectively. The aldosterone-to-cortisol ratio was 340 times higher in her right adrenal vein than in her left adrenal vein. These findings demonstrated that her right adrenal gland was the source of excess aldosterone secretion.

Our patient was informed about the details of the surgical procedure and our efforts to reduce the number of incisions needed - ideally, to a single incision - when removing her adrenal gland. A SPLA was attempted.

Our patient was placed in the left semilateral position. A 2.5cm incision was made on the umbilicus, and a multichannel port (SILS™ port; Covidien, Mansfield, MA, USA) was inserted ( 2). The pneumoperitoneal pressure was maintained at 10mmHg. Three 5mm cannulas were inserted through the SILS™ port. The primary tools used in the operation were a 5mm flexible laparoscope (Olympus Medical Systems, Tokyo, Japan), a roticulated laparoscopic dissector (Covidien) and a tissue sealing device (ENSEAL®; Ethicon, Cincinnati, OH, USA). Her right liver lobe was evaluated using a percutaneous instrument (MiniLap; Stryker, Kalamazoo, MI, USA), providing good visualization of the operative field surrounding the right adrenal gland (Figure 3). The overall procedure was similar to the procedure performed in a conventional laparoscopic anterior adrenalectomy using a four-port technique. Only her right central adrenal vein was clipped and the small adrenal vessels were divided using an ENSEAL®. Her adrenal gland was extracted via a retrieval bag by removing one 5mm cannula and upsizing to a 12mm cannula. The skin was closed with absorbable sutures. No drains were inserted.

**Figure 2:**
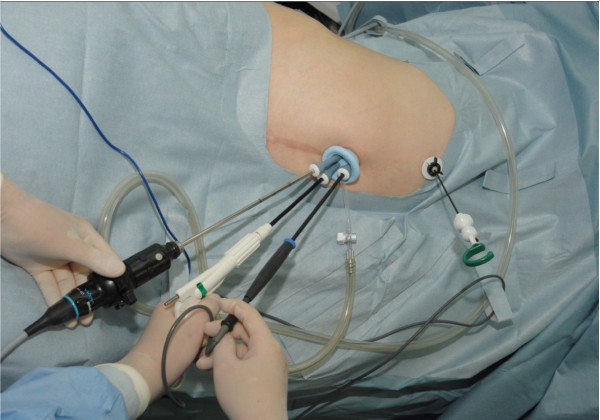
**Port placement for right single-port laparoscopic adrenalectomy.** The SILS™ port was placed through a 2.5cm umbilical skin incision.

**Figure 3:**
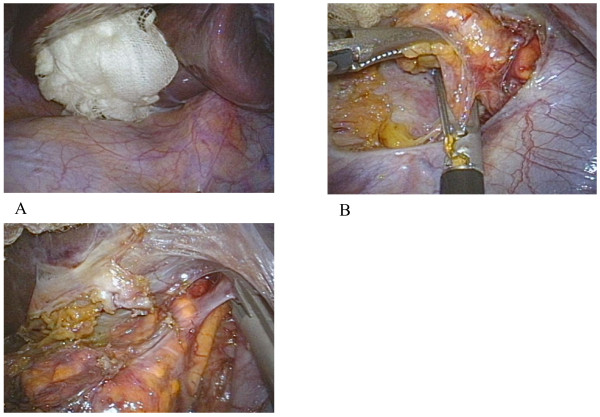
**Intraoperative findings. (a)** Good visualization of the operative field using a MiniLap. **(b)** Adrenal dissection using a tissue sealing device. **(c)** Clipping of the right central adrenal vein.

SPLA was successfully completed without the need for any skin incisions or additional ports. The operating time was 76 minutes, and her blood loss was 5mL. Oral intake was resumed on the first postoperative day, and the length of her hospital stay was three days. Pathological examination confirmed a cortical adenoma of the adrenal gland. No surgical site infections or incisional hernias were noted during outpatient follow-up. Our patient has had an excellent cosmetic result on postoperative follow-up.

## Discussion

A conventional MPLA using three or four ports is the gold standard operative treatment for benign adrenal diseases. The advantages of an MPLA include decreased scarring, decreased incisional pain, shorter hospitalization and faster functional recovery. Generally, the goal has been to minimize the invasiveness of this procedure by reducing the number or size of the operating ports. Since March 2009, we have been using single-port laparoscopic cholecystectomies in selected patients with benign gallbladder diseases. In addition, our team has recently performed successful advanced SPSs, such as gastrectomy, splenectomy, left adrenalectomy, Heller-Dor procedure and Nissen fundoplication [9-12].

In 2008, Castellucci *et al*. [13] reported the first SPLA in a 63-year-old female patient with a 4.5cm left adrenal incidentaloma. They used a three-port technique, introduced through a 2.5cm supra-umbilical incision and successfully removed a pheochromocytoma. However, SPLA is still limited by the surgical team’s adrenal and laparoscopic experience [14-17]. At our institution, SPLA was introduced after more than 80 adrenalectomies were conducted using a laparoscopic approach. This report documents our first patient to undergo a SPLA through her umbilicus for a right-sided adrenal tumor. The most important technical challenge for right-sided SPLA is providing a good operative field surrounding the right adrenal gland. However, elevation of the right liver lobe using a MiniLap provided good visualization of the operative field, which reproduced a similar MPLA. The assistance of the needlescopic instrument does not compromise the cosmetic outcome; this outcome is still considered to be one of the main advantages of SPLA over MPLA.

In our experience, with 27 conventional MPLAs for patients with right-sided aldosterone-producing adenomas, the median operating time was 90 minutes (range, 60 to 155 minutes) and the median blood loss was 5mL (range, 1 to 57mL). No differences were noted in operative outcomes between SPLA and MPLA. In the future, we believe that this approach for treating a right-sided adrenal tumor might be one of the next frontiers of laparoscopic surgery because of the benefits gained from using SPS, such as higher cosmesis satisfaction in patients and the lack of differences in operative outcomes between SPLA and MPLA.

## Conclusions

Based on our experience with this case, we believe that SPLA is a safe and feasible procedure for patients with a right-sided small adrenal tumor when performed by a surgeon experienced in laparoscopic and adrenal surgery. However, more surgical experience using this technique is required to confirm our initial impressions.

## Abbreviations

MPLA, Multiport laparoscopic adrenalectomy; SPLA, Single-port laparoscopic adrenalectomy; SPS, Single-port surgery.

## Consent

Written informed consent was obtained from the patient for publication of this case report and any accompanying images. A copy of the written consent is available for review by the Editor-in Chief of this journal.

## Competing interests

The authors declare that they have no competing interests.

## Authors’ contributions

AS drafted the first manuscript. AS, SB, TO, AU and MM cared for the patient. GW helped to draft the manuscript. All authors read and approved the final manuscript.
